# Significance of Crooke's Hyaline Change in Nontumorous Corticotrophs of Patients With Cushing Disease

**DOI:** 10.3389/fendo.2021.620005

**Published:** 2021-03-18

**Authors:** Amit Akirov, Vincent Larouche, Ilan Shimon, Sylvia L. Asa, Ozgur Mete, Anna M. Sawka, Fred Gentili, Shereen Ezzat

**Affiliations:** ^1^ Department of Endocrine Oncology, Princess Margaret Cancer Centre, University of Toronto, Toronto, ON, Canada; ^2^ Institute of Endocrinology, Beilinson Hospital, Petach Tikva, Israel; ^3^ Sackler School of Medicine, Tel Aviv University, Tel Aviv, Israel; ^4^ Division of Endocrinology and Metabolism, Jewish General Hospital, McGill University, Montreal, QC, Canada; ^5^ Department of Pathology, University Hospitals, Cleveland, Case Western Reserve University, Cleveland, OH, United States; ^6^ Department of Pathology, University Health Network, University of Toronto, Toronto, ON, Canada; ^7^ Division of Endocrinology, University Health Network and University of Toronto, Toronto, ON, Canada; ^8^ Division of Neurosurgery, Toronto Western Hospital, University Health Network and Department of Surgery, University of Toronto, Toronto, ON, Canada

**Keywords:** Cushing disease, corticotroph tumor, Crooke's changes, pituitary tumors, pituitary tumor regrowth and recurrence

## Abstract

**Background:**

Glucocorticoid excess in Cushing disease (CD) leads to negative feedback suppression, resulting in Crooke's hyaline change (CC) of nontumorous pituitary corticotrophs. We aimed to determine the predictive value of CC of nontumorous corticotrophs in CD.

**Methods:**

The retrospective chart review study included patients with clinical, biochemical, radiologic and outcome data and evaluable histopathology specimens from pituitary surgery for CD. The main outcome was remission of CD, defined by clinical features, biochemical testing, and corticosteroid dependency.

**Results:**

Of 144 CD patients, 60 (50 women, mean age 43.6±14) had clinical follow-up, biochemical data and histopathology specimens that included evaluable nontumorous adenohypophysis. Specimens from 50 patients (83.3%) demonstrated CC in nontumorous corticotrophs, and 10 (16.7%) had no CC (including 3 with corticotroph hyperplasia). One patient with CC was lost to follow-up and one without CC had equivocal outcome results. During a mean (SD) follow-up period of 74.9 months (61.0), recurrent or persistent disease was documented in 18 patients (31.0%), while 40 (69.0%) were in remission. In patients with CC, the remission rate was 73.5% (95% CI, 59.7%-83.7%) (36/49), whereas it was 44.4% (95% CI, 18.9%-73.3%) (4/9) in patients with no CC. The combination of serum cortisol >138 nmol/L within a week of surgery coupled with absence of nontumorous CC greatly improved the prediction of recurrent or persistent disease.

**Conclusions:**

CC of nontumorous corticotrophs was observed in 83% of patients with CD, and most patients with CC experienced remission. Absence of CC in nontumorous corticotrophs may serve as a predictor of reduced remission in patients with CD.

## Introduction 

**Figure 1:**
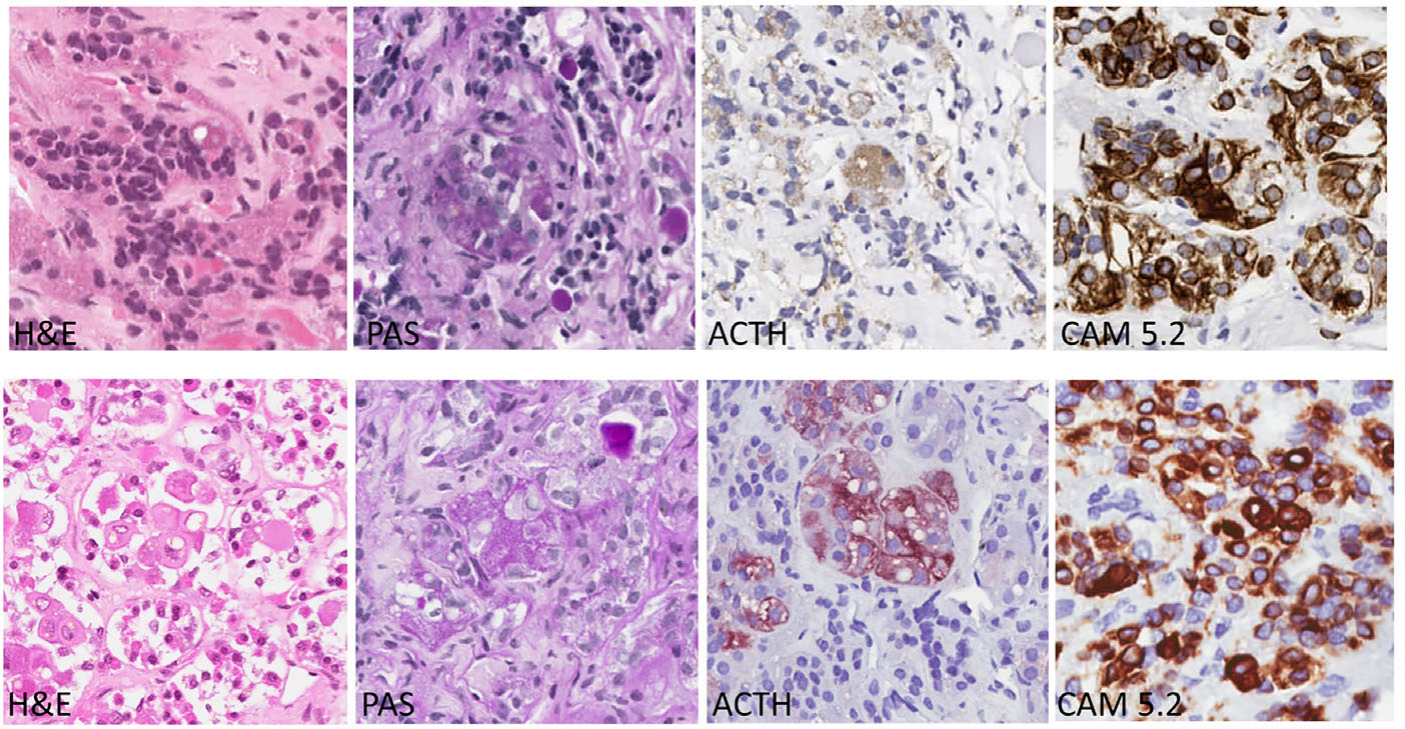
Corticotroph Cells with Crooke’s Hyaline Changes (Bottom row) Compared with Normal Corticotrophs (Top Row). Crooke’s hyaline has a pale acidophilic appearance in slides stained with hematoxylin and eosin (H&E); the corticotrophs with this change have large clear lysosomes known as enigmatic bodies. With the periodic acid Schiff (PAS) stain, the granules of corticotrophs with Crooke’s hyaline change are found only at the cell periphery or in a juxtanuclear location. The same pattern is seen in stains for ACTH. In contrast, the hyaline material is strongly positive with the CAM 5.2 stain that localizes the keratin filaments as thick, intensely-stained bands filling the cytoplasm.

Glucocorticoid excess leads to negative feedback suppression, resulting in Crooke's hyaline change (CC) of normal corticotrophs in the pituitary. Crooke’s cells are corticotrophs with concentric cytoplasmic accumulation of cytokeratin filaments. This accumulation of glassy, homogeneous, hyaline material in the cytoplasm leads to displacement of the secretory granules around the nucleus or at the periphery of the cytoplasm. Immunostaining for CAM5.2 can show strong reactivity of the Crooke's cells (1–3) (**Figure 1**). This change is identified in the pituitaries of patients with any source of glucocorticoid excess, including Cushing syndrome due to exogenous corticosteroids, adrenal cortical tumors, and ectopic ACTH syndrome. In patients with Cushing disease (CD), this change may be seen in the nontumorous pituitary tissue in response to glucocorticoid excess resulting from an ACTH-producing pituitary corticotroph tumor (4–7).

The treatment of choice for Cushing's disease is transsphenoidal surgery with removal of the pituitary tumor (8). There is a wide variation in persistence or recurrence rates following pituitary surgery for ACTH-producing pituitary tumors, ranging between 7-46% with higher recurrence rates following surgical treatment for macrotumor compared to microtumor (9–14). Other than the size of the lesion, additional factors that may aid in predicting remission include clear evidence of pituitary tumor on imaging and the neurosurgeon's experience (15, 16).

No consensus has been established regarding the criteria for remission following surgery to remove an ACTH-producing pituitary tumor. In the clinical practice guidelines for treatment of CD by the Endocrine Society, remission is defined as serum cortisol <138 nmol/L or 24-hour urinary free cortisol <28-56 nmol/d within a week of pituitary surgery (8). Frequently, the need for glucocorticoid requirement is used to establish remission of CD following surgery. However, as the hypothalamic-pituitary-adrenal axis (HPA) may recover with time, it is recommended to evaluate patients for possible CD recurrence. Following HPA recovery, resolution of symptoms along with various constellations of biochemical testing are used to determine or predict recurrent disease. Biochemical testing for recurrence may include late-night salivary cortisol, 24-hour urinary free cortisol, and/or dexamethasone suppression test. Combined testing with CRH or desmopressin can also assist in predicting disease recurrence (8). As mortality rates are significantly higher in patients with persistent or recurrent hypercortisolism (17–19) which may develop many years after successful pituitary surgery, it is recommended to continue surveillance for CD recurrence throughout life (8).

Limited data are available regarding the significance of the presence or absence of Crooke’s hyaline changes of the nontumorous corticotrophs in patients with CD. Our goal was to determine the prevalence and the implications of finding of CC on histopathology examination. 

## Methods

This retrospective chart review study was conducted using a study population from an Endocrine Oncology clinic (SE) in a large University-affiliated tertiary- and quaternary care medical center (University Health Network in Toronto, Canada). The patients were identified through review of billing diagnostic codes and pathology records.

### Study Inclusion and Exclusion Criteria

We reviewed the electronic medical records of all adult (≥18 years of age) patients with a diagnosis of CD before surgery, who had pituitary surgery between January 2001 and December 2018. The diagnosis of CD was based on elevated 24-h urinary free cortisol, failure to suppress serum cortisol after low-dose dexamethasone testing, radiographic, and histological confirmation of a pituitary source of hormone excess. The pathology specimens were reviewed by two expert endocrine pathologists (SLA and OM), for the presence of CC in the nontumorous adenohypophysis. The preoperative diagnosis of CD was confirmed following pituitary surgery, based on the pathology review of the specimen. We included patients who had more than one pituitary surgery, but focused on their last pituitary surgery for our analysis. When possible, we also reviewed the results of evaluation of CC in samples from previous surgeries. We excluded patients with incomplete clinical, biochemical and radiological data, patients with non-pituitary Cushing syndrome, patients with unavailable pathology sample for review, as well as histopathological specimens that lacked sufficient nontumorous adenohypophysis and those with tissue handling artifacts that precluded reliable morphological assessment of the nontumorous corticotrophs. Patients lost to follow-up were excluded from the statistical analyses.

### Histopathology Review and Definitions

The presence or absence of CC was determined based on histopathology analysis of the nontumorous corticotrophs in the normal pituitary gland, obtained during surgical intervention to resect the ACTH-producing pituitary corticotroph tumor. Corticotroph tumors were classified as densely granulated or sparsely granulated corticotroph tumors based on the cytoplasmic intensity of staining with hematoxylin (basophilic vs chromophobic), with the PAS stain that decorates glycoproteins and ACTH immunohistochemistry that localizes ACTH in the cytoplasm. EM was not performed in these cases as it is not routine (7). Tumor invasion was reported based on the details of the radiologic investigations. There is no value in determining histological invasion as it is seen in the majority of pituitary tumors (3, 20).

### Data Collection and Entry

We (AA,VL) collected demographic, clinical, biochemical and imaging data from the electronic medical record and clinical chart. Data was electronically entered in Excel. The study was approved by the University Health Network Research Ethics Board. Written, informed patient consent was not needed for this retrospective study.

### Definition of Clinical Outcomes

The primary outcome was the final CD status, including remission, recurrence, and persistent disease. These were described according to presence or absence of CC in the nontumorous pituitary. Remission of CD was defined at last follow-up visit, according to the following: (a) clinical signs and symptoms; (b) biochemical testing, including 24-hour urinary free cortisol, overnight 1-mg dexamethasone suppression test, or salivary cortisol, as well as ACTH levels; (c) evidence for hypocortisolism and corticosteroid dependency. Persistent disease was defined when there was evidence for CD on clinical and/or biochemical studies following pituitary surgery. Recurrent disease was defined when there was evidence of remission, as defined above, but subsequent recurrence of clinical and biochemical findings of CD.

### Statistical Analyses

We summarized the data descriptively, including numbers and percentages for categorical data and mean or median and standard deviation (SD) or range for continuous data. For the primary outcome, we calculated 95% confidence intervals (CI) for the percentage of patients according to disease status and presence or absence of CC in the nontumorous adenohypophysis (Wilson’s methods, Confidence Interval Analysis Software, Version 2.0.0).

## Results

### Study Population

**Figure 2:**
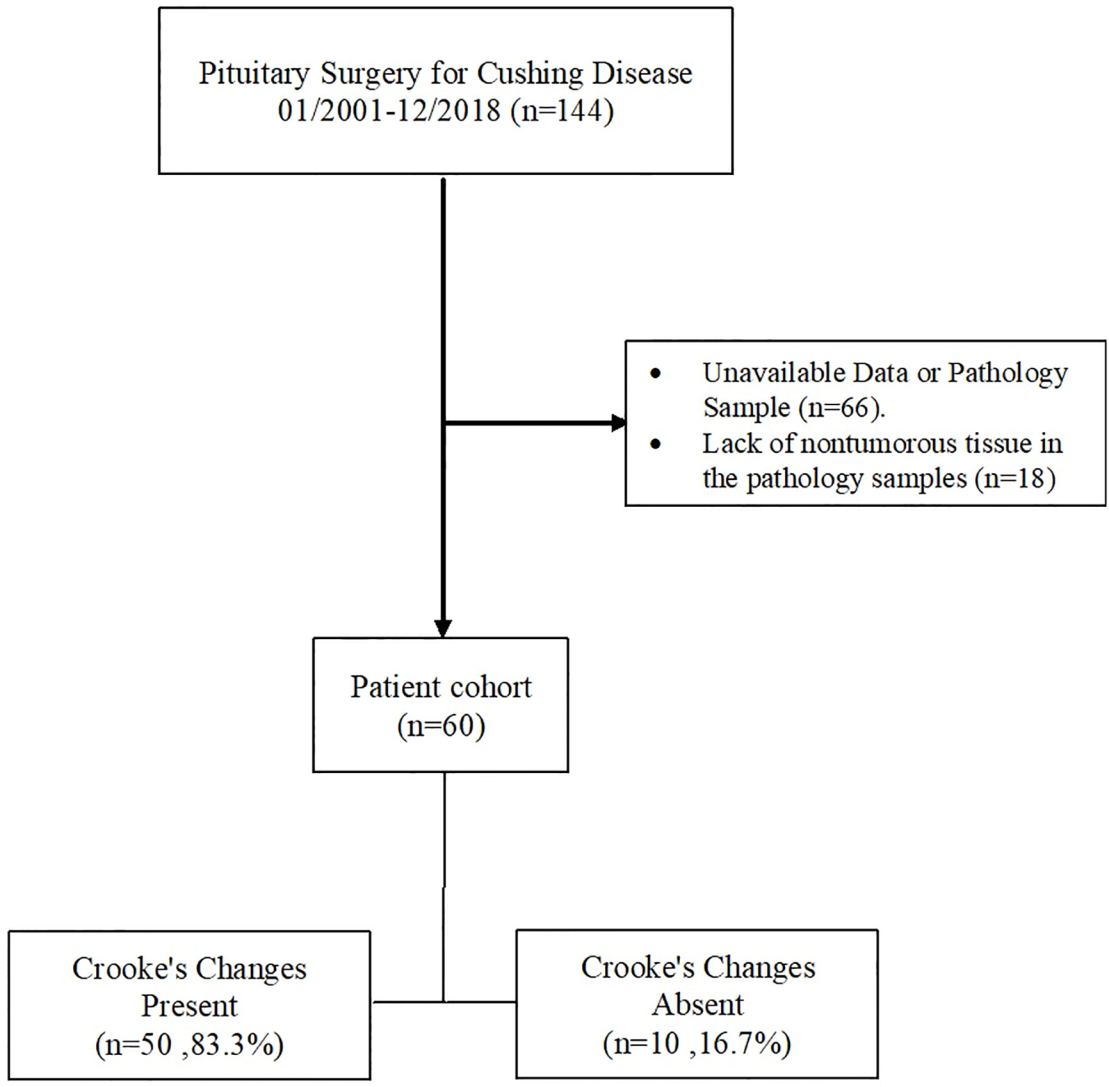
Flow diagram of exclusion criteria to arrive at the final analysis profile. The records of all patients who had pituitary surgery between January 2001 and December 2018 were screened as described in the text.

Of 144 patients who had pituitary surgery for CD, 66 were excluded due to lack of a pathology sample that was reviewed by an expert pathologist or lack of complete clinical data, including biochemical and imaging studies to confirm the clinical diagnosis of CD or remission vs. recurrent/persistent disease. All excluded patients (mean age, 44.1 ± 15.7 years; 51 women) had a diagnosis of CD in their medical chart, data on pituitary imaging was only available for 12 patients (mean tumor size 7.8 mm, 3 patients with macrotumors), and densely granulated corticotroph tumor was the most common histological type (40 cases, 61%), followed by Crooke cell adenoma (10 cases, 15%), mixed densely granulated and sparsely granulated tumor (5 cases, 8%), and sparsely granulated tumor (4 cases, 6%). Of the remaining 78 CD patients, evaluation of CC in the adjacent adenohypophysial tissue was not possible in 18 (mean age 45.0 ± 13.0 years; 14 women) patients (20 pathology samples), mainly due to insufficient nontumorous pituitary tissue (13 samples), crush artifact (4 samples), or other reasons (3 samples) (**Figure 2**).

The study sample therefore included 60 patients (50 women, mean age 43.6 ± 14) with clinical and biochemical evidence of CD before pituitary surgery, and positive PAS and ACTH-staining corticotroph tumors on histologic examination of surgically resected tissue specimens. All patients had at least one available pathology sample for evaluation; 17 patients had more than one available sample. Most patients had an intrasellar (55 patients, 91.7%) microtumor (52 patients, 86.7%). Suprasellar extension and/or cavernous sinus invasion were reported in 5 patients (8.3%) (**Table 1**).

**Table 1:** Cohort Characteristics.

	Nontumorous Crooke’s Changes (+)	Nontumorous Crooke’s Changes’ (-)
** **	(N=50)	(N=10)
** **		
**Clinical Features**		
**Age, average**	43±15	48±11
**Male, n (%)**	8 (16%)	2 (20%)
**Diabetes, n (%)**	20 (41%)	2 (20%)
**Hypertension, n (%)**	27 (55%)	8 (80%)
**Cardiomyopathy, n (%)**	3 (6%)	0 (0%)
**Skeletal Disease, n (%)**	9 (18%)	2 (20%)
		
**Imaging Features**		
**Tumor Size**		
**Microtumor, n (%)**	44 (88%)	8 (80%)
**Macrotumor, n (%)**	6 (12%)	2 (20%)
Tumor Location*		
**Right Side, n (%)**	25 (50%)	5 (50%)
**Central, n (%)**	5 (10%)	1 (10%)
**Left Side, n (%)**	21 (42%)	5 (50%)
Tumor Invasion**		
**Intrasellar, n (%)**	47 (94%)	8 (80%)
**Suprasellar, n (%)**	3 (6%)	2 (20%)
**Cavernous Sinus Invasion, n (%)**	3 (6%)	2 (20%)

*Pituitary tumor involving both right and left pituitary in two cases (one case with nontumorous CC+ and one case of CC-) and one case involving the right, left and central pituitary (in the group with no data regarding CC).

**Pituitary tumor involving invasion of both suprasellar structures and cavernous sinus in three cases.

The clinical features and imaging characteristics of patients with and without evidence of Crooke’s hyaline changes and patients with no data regarding Crooke’s hyaline changes.

Among these 60 patients with adequate pathology samples for evaluation of nontumorous CC using cytomorphologic and immunohistochemical biomarkers (CAM5.2, ACTH and PAS), in 50 patients (83.3%) histologic examination of the surgical specimen revealed CC in the adjacent nontumorous adenohypophysial tissue. In 10 patients (16.7%), the samples showed lack of CC; in 3 of these there was evidence of corticotroph hyperplasia.

There was no difference in the age or gender between patients with CC (mean age±SD, 43±15, 84% female) and those without CC (mean age 48±11,80% female) (**Table 1**).

Following pituitary surgery, only one patient developed persistent diabetes insipidus, none received treatment with growth hormone or gonadal steroids, and 7 patients were treated for hypothyroidism.

### Persistent, Recurrence, and Remission

During a mean (SD) follow-up period of 74.9 months (61.0), recurrent or persistent disease was documented in 18 patients (31.0%), while 40 patients (69.0%) were in remission. One patient with nontumorous CC was lost to follow-up and in another patient without nontumorous CC the outcome was indeterminate due to equivocal results.

Remission rates in patients with no histological evidence of CC in the nontumorous corticotrophs were 44.4%; 18.9%-73.3% (4 of 9 patients) and those with evidence of nontumorous CC were 73.5%; 95% CI, 59.7%-83.7% (36 of 49 patients) (**Table 2**). In the group of patients with inadequate pathology for histologic examination of nontumorous CC, 50.0% of patients remained in remission (9/18 patients) (**Table 2**).

**Table 2:** Biochemical and Radiological Outcomes.

	Patients	Cortisol < 138 nmol/L within 7 days of surgery			Normal ACTH within 30 days of surgery			Normal MRI at the end of follow-up			Definition of Remission	
		Yes	No	NA	Yes	No	NA	Yes	No	NA	Glucocorticoid Dependency	Normal 24-h UFC*
**CC+, Remission**	36	26 (72%)	6 (17%)	4 (11%)	23 (61%)	–	13 (39%)	28 (78%)	4 (11%)	4 (11%)	9 (25%)	27 (75%)
**CC+, Persistence/Recurrence**	13	2 (15%)	9 (69%)	2 (15%)	8 (62%)	4 (31%)	1 (8%)	6 (46%)	6 (46%)	1 (8%)	–	–
**CC-, Remission**	4	3 (75%)	1 (25%)	–	1 (25%)	–	3 (75%)	3 (75%)	–	1 (25%)	2 (33%)	2 (33%)
**CC-, Persistence/Recurrence**	5	–	5 (100%)	–	4 (80%)	0 (50%)	1 (20%)	–	5 (100%)	–	–	–

*UFC, urinary free cortisol.

Remission or persistent/recurrent disease according to presence or absence of Crooke’s hyaline changes based on serum or urine cortisol levels, serum ACTH levels, imaging studies and glucocorticoid dependence.

Remission following pituitary surgery was achieved in 40 patients with no clinical or biochemical evidence of CD recurrence. Remission was defined based on glucocorticoid dependency in 12 patients, including 9 cases in the group of patients with CC, and 2 subjects with no histological evidence of CC. In 29 patients, 24-hour urinary free cortisol was normal, including 27 of those with histologic evidence of CC and 2 with no evidence of CC. Of all patients in remission, imaging was available and showed no evidence of tumor in 31 patients, while in 4 patients there was evidence of non-specific post-operative changes, and in 5 patients, MRI data were not available (**Table 2**).

Of all 40 patients in remission at the end of follow-up, cortisol levels within 7 days of pituitary surgery were available for 36 patients, with levels <138 nmol/L in 29 patients (80.6%), and higher than this cutoff in 7 patients (17.5%). Of those 7 patients with serum cortisol levels >138 nmol/L following surgery that went into remission, all except one patient had CC in the nontumorous tissue (**Table 2**).

Of all 49 patients with evidence of CC in the nontumorous corticotrophs, serum cortisol levels within 7 days of pituitary surgery were available for 28 patients. In this group, cortisol levels were < 138 nmol/L in 26 of 36 (72%) patients in remission and in 2 of 13 patients (15%) with persistent/recurrent disease (**Table 2**).

Low serum cortisol levels within one week of pituitary surgery were observed in 80.6% of patients with available data (29 of 36 patients) and in remission at the end of follow-up. Remission was evident in 73.5% of patients with evidence of CC (36/49 patients), and the presence of CC in addition to low serum cortisol levels improved the prediction rate for remission (93%; 26 of 28 patients with evidence of CC in the nontumorous corticotrophs and low serum cortisol were in remission at the end of follow-up).

Most patients (14/21, 66.7%) who had high serum cortisol during the first week following surgery had recurrent/persistent disease at the end of follow-up. Among patients with evidence of CC in the nontumorous corticotrophs and high serum cortisol levels, 60.0% (9/15 patients) had persistent/recurrent disease. Among those with no evidence of CC in the nontumorous corticotrophs and high serum cortisol levels, 83.4% (5/6 patients) had persistent/recurrent disease. All patients with persistent/recurrent disease at the end of follow-up and no evidence of CC had high serum cortisol during the first week following surgery (5/5) (**Table 2**).

Of the 18 patients excluded from the analysis due to inadequate pathology for histologic examination of CC (8 patients with a macrotumor), 50% of patients remained in remission (9/18 patients).

### Pituitary Tumor Characteristics

In the group of patients with evidence of CC in the nontumorous corticotrophs, most of the pituitary tumors were intrasellar tumors (47/50 patients, 94%), and in those without CC, 80% of the cases were intrasellar tumors (8/10 patients) (**Table 1**).

In the group of patients with CC in the nontumorous corticotrophs, macrotumor was evident in 6 cases and in 5 patients pituitary surgery led to remission. Two patients had evidence of suprasellar extension and cavernous sinus invasion, one went into remission following surgery, and the other had persistent/recurrent disease. Another patient had evidence of cavernous sinus infection with no suprasellar extension, and had persistent/recurrent disease.

In those with no evidence of CC, a macrotumor was found in 2 patients, and surgery led to remission in one and did not lead to remission and in the other patient that required bilateral adrenalectomy. Two patients had evidence of suprasellar extension with cavernous sinus invasion, and both had persistent/recurrent disease following surgery, as was another patient with cavernous sinus invasion without suprasellar extension.

More than half of the tumors were densely granulated corticotroph tumors (39/60, 65.0%), the remainder included sparsely granulated (6/60, 10%) or mixed sparsely and densely granulated corticotroph tumors (4/60, 6.7%). Crooke cell tumor (pituitary corticotroph tumor with prominent Crooke cell change) was evident in 5 cases (8.3%), and corticotroph hyperplasia with no discrete tumor in 3 patients (5.0%), with additional suspected case of corticotroph hyperplasia.

### Additional Pituitary Surgeries

Most patients had one pituitary surgery (37 patients, 61.7%), while a third (19 patients, 31.7%) required two operations, and only a smaller group (4 patients; 6.7%) required more than two surgeries While our study focused on the results following the last pituitary surgery, pathology specimens of previous surgeries were available in 19 of these cases. In almost all cases (14 of 18 patients), the presence or absence of CC in the nontumorous pituitary was concordant in all samples.

The group of patients with evidence of CC in the nontumorous adenohypophysis included 18 patients with at least one additional previous pituitary surgery (16 patients had a total of two pituitary surgeries, 2 patients had three pituitary surgeries). Of these 18 patients, initial pathology specimen was available for 13 patients, including 10 patients with evidence of nontumorous CC in the specimen from the previous surgery, of whom 4 went into remission after the second transsphenoidal surgery, and 6 had persistent/recurrent disease. In 2 cases where CC was not reported due to lack of nontumorous tissue in the initial pathology samples, following the second surgery there was evidence of CC and the patients went into remission. In an additional patient there was no evidence of CC on the initial histologic evaluation, and this patient had persistent disease after the second surgery and eventually required bilateral adrenalectomy.

The group of patients without evidence of nontumorous CC included 5 patients with at least one additional previous pituitary surgery (3 patients had a total of two pituitary surgeries and 2 patients had three pituitary surgeries). Of these 5 patients, the initial pathology sample was available for 4 patients, and in 3 cases there was no evidence of nontumorous CC in the initial specimen as well.

## Discussion

Our study assessed outcome of surgical intervention for CD according to the presence or absence of CC in the nontumorous corticotrophs of patients with CD. We observed that in cases with an adequate pathology sample, examined by experienced pathologists for the presence of CC in the nontumorous corticotrophs, these can be found in over 80% of patients with CD. Our data suggest that in cases where there was evidence for nontumorous CC, remission rates were >70%, while in the absence of nontumorous CC, persistent/recurrent disease was documented in <50% of patients. Furthermore, while low serum cortisol levels within one week of pituitary surgery were predictive of remission, the combination of serum cortisol >138 nmol/L within a week of surgery coupled with absence of nontumorous CC greatly improved the prediction of recurrent or persistent disease.

Evidence of CC in the nontumorous corticotrophs confirms the diagnosis of pathologic glucocorticoid excess, thus supports the diagnosis of CD and enables the distinction of pseudo-Cushing, even when a pituitary tumor is not identified on pathologic examination, as may occur with very tiny tumors (5, 7, 21). However, in the absence of a corticotroph tumor, as shown by Pouratian et al., the presence of CC confirms that the patient has raised circulating glucocorticoids, but this could be secondary to causes other than CD, such as ectopic ACTH secretion, primary cortisol-producing adrenal tumor, or exogenous glucocorticoid use. The absence of CC in nontumorous corticotrophs of a patient with no detectable tumor usually indicates a misdiagnosis of Cushing syndrome (i.e. a pseudo-Cushing phenomenon); it may, in rare cases, represent a failure to resect a tumor in a patient who does not have normal feedback suppression, however as our data show, this occurs in only 16.7% of patients and at least 30% of those have diffuse corticotroph hyperplasia that would be diagnosed in the sample resected (22). Previously, it was reported that CC depends on the degree of hypercortisolism, and in a study by Oldfield et al., almost all patients with maximum 24-hour urinary free cortisol that was at least 4-fold greater than the upper limit of normal had nontumorous CC (1).

The results of our study underscored the clinical importance of nontumorous CC, in considering long term outcomes of patients with CD. Hague et al. collected data of 21 patients with CD following resection of intrasellar microtumor that were reviewed by two pathologists for the presence of CC in the nontumorous adenohypophysis. Their study reported that the absence of CC in peritumoral pituitary was associated with unexpected recurrence of CD, as all three patients with no evidence of CC had persistent/recurrent disease, as did 4 of 18 patients (22%) with evidence of CC (4). In contrast, Cordeiro et al. reported that in a series of 61 CD patients treated by gamma knife radiosurgery, there was no difference in remission rates in the presence of CC (76.9%) or absence (81.8%) of CC (23); this may be attributed to radiation effects on the nontumorous adenohypophysis. Our data and that of Hague et al. indicate that examination directed specifically at the detection of nontumorous CC may on one hand aid in confirming the diagnosis of negative feedback regulation of CD, and on the other hand, point to greater risk of persistent or recurrent disease.

Previous studies have reported conflicting results as to the prevalence of nontumorous CC in patients with CD. While one German registry reported that in all adequate samples of ACTH-secreting pituitary tumor there was evidence of nontumorous CC (24), others reported that nontumorous CC was found in less than 40% of patients (25). Our results are in line with those reported by Oldfield et al. that identified CC in 74% of patients who had pituitary CD (1).

While the prevalence of CC was very high in cases of intrasellar microtumors, evaluation of CC in the nontumorous adenohypohysis might be more limited in cases of macrotumors; in most cases of macrotumor in our cohort, evaluation of CC was not technically possible, mainly due to insufficient nontumorous gland in the specimen. This may be the result of tumor destruction of the nontumorous pituitary, the more likely requirement for aggressive suction during surgery resulting in loss of tissue, and/or different sampling by the neurosurgeon when selecting tissue for pathology. Nevertheless, when evaluation of CC was possible in cases of macrotumor, the presence of CC was favorably associated with remission.

Similar to previous reports, post-operative ACTH levels shortly after pituitary surgery were not a good predictor of remission (15). However, our results support the Endocrine Society guidelines for the use of serum cortisol <138 nmol/L within a week of pituitary surgery to predict remission (8), as >80% of our cohort with low serum cortisol were in remission at the end of follow-up and addition of the nontumorous CC to low serum cortisol levels only slightly improved the prediction rate for remission. This is similar to a previous report by Costenaro and colleagues, reporting cortisol nadir ≤ 157 ng/mL within 10-12 days of pituitary surgery had a sensitivity of 91% and specificity of 100% for CD remission (26).

On the other hand, this serum cortisol cutoff was not as good in predicting persistent/recurrent disease, as only two-third of patients with serum cortisol >138 nmol/L had recurrent/persistent disease. These findings are in line with a previous report by Rollin and colleagues, reporting that not all patients with surgically induced remission had low postoperative serum cortisol. They suggested cortisol levels do not decrease immediately after surgical intervention and several days may be necessary for cortisol to reach low levels (27). Simmons et al. suggested to obtain midnight preoperative serum cortisol levels and compare these with post-surgical cortisol levels before introducing corticosteroid treatment, as the individual cortisol dynamics may predict outcome and potentially identify patients who may experience a delayed disease remission (28). In our study, the assessment of nontumorous CC on histopathology appeared helpful in identifying patients at high risk for persistent/recurrent disease.

Our study had several limitations. The retrospective design of the study and the small sample size limit the power of the study and precluded any meaningful direct statistical comparisons of group. The heterogeneous group of patients, that included cases of micro- and macro-tumor, single and multiple pituitary surgeries, is another possible limitation of the study. Furthermore, we did not have serum cortisol levels within a week of pituitary surgery for all cases. The strengths of the study include the evaluation of CC in the nontumorous pituitary gland by two expert pathologists; studying the predictive value of the nontumorous CC, which was not extensively investigated previously; and investigating for the first time the correlation of CC in the nontumorous pituitary sample with serum cortisol levels shortly after pituitary surgery, resulting in our novel finding that the combination of high serum cortisol levels with absence of nontumorous CC is highly predictive of persistent/recurrent disease.

In conclusion, in CD patients treated surgically, evaluation of CC in the nontumorous pituitary gland may be valuable, as absence of CC may point to increased risk of persistent or recurrent disease after pituitary surgery.

## Data Availability Statement

The raw data supporting the conclusions of this article will be made available by the authors, without undue reservation.

## Ethics Statement

The studies involving human participants were reviewed and approved by University Health Network Research Ethics Board. Written informed consent for participation was not required for this study in accordance with the national legislation and the institutional requirement.

## Author Contributions

AA: substantial contributions to conception and design, acquisition of data or analysis and interpretation of data, drafting the article or revising it critically for important intellectual content, final approval of the version to be published. VL: substantial contributions to conception and design, acquisition of data or analysis and interpretation of data, drafting the article or revising it critically for important intellectual content, final approval of the version to be published. IS: substantial contributions to conception and design, acquisition of data or analysis and interpretation of data, drafting the article or revising it critically for important intellectual content, final approval of the version to be published. SA: substantial contributions to conception and design, acquisition of data or analysis and interpretation of data, drafting the article or revising it critically for important intellectual content, final approval of the version to be published. OM: substantial contributions to conception and design, acquisition of data or analysis and interpretation of data, drafting the article or revising it critically for important intellectual content, final approval of the version to be published. AS: substantial contributions to conception and design, acquisition of data or analysis and interpretation of data, drafting the article or revising it critically for important intellectual content, final approval of the version to be published. FG: substantial contributions to conception and design, acquisition of data or analysis and interpretation of data, drafting the article or revising it critically for important intellectual content, final approval of the version to be published. SE: substantial contributions to conception and design, acquisition of data or analysis and interpretation of data, drafting the article or revising it critically for important intellectual content, final approval of the version to be published. All authors contributed to the article and approved the submitted version.

## Conflict of Interest

The authors declare that the research was conducted in the absence of any commercial or financial relationships that could be construed as a potential conflict of interest.
